# 4-[(*E*)-(3-Methyl-5-thioxo-4,5-dihydro-1*H*-1,2,4-triazol-4-yl)imino­meth­yl]benzonitrile

**DOI:** 10.1107/S1600536808017613

**Published:** 2008-06-13

**Authors:** Yu-Yuan Zhao, Hong Zhao, Wen-Xiang Wang, Jie Xiao

**Affiliations:** a Ordered Matter Science Research Center, College of Chemistry and Chemical Engineering, Southeast University, Nanjing 210096, People’s Republic of China

## Abstract

In the title compound, C_11_H_9_N_5_S, the dihedral angle between the mean planes of the thione-substituted triazole ring and benzonitrile ring is 4.28 (3)°. Inter­molecular N—H⋯S hydrogen bonds link the mol­ecules together into characteristic dimers.

## Related literature

For the application of benzotriazole compounds in industry, see: Sharma & Bahel (1982 ▶); Grasso (1988 ▶); Eweiss *et al.* (1986 ▶); Awad *et al.* (1991 ▶); Pillard *et al.* (2001 ▶). For bond-length data, see: Allen *et al.* (1987 ▶).
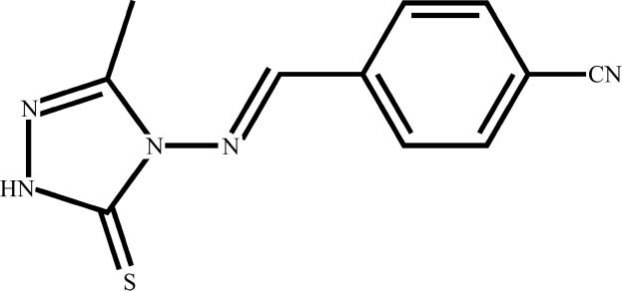


## Experimental

### 

#### Crystal data

C_11_H_9_N_5_S
*M* *_r_* = 243.29
Triclinic, 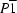

*a* = 6.975 (2) Å
*b* = 7.682 (2) Å
*c* = 11.412 (2) Å
α = 90.262 (7)°
β = 94.328 (14)°
γ = 104.713 (17)°
*V* = 589.5 (3) Å^3^
*Z* = 2
Mo *K*α radiation
μ = 0.26 mm^−1^
*T* = 293 (2) K
0.70 × 0.50 × 0.50 mm

#### Data collection

Rigaku Mercury2 diffractometer
Absorption correction: multi-scan (*CrystalClear*; Rigaku, 2005 ▶) *T*_min_ = 0.854, *T*_max_ = 0.901
5954 measured reflections
2659 independent reflections
2178 reflections with *I* > 2σ(*I*)
*R*_int_ = 0.022

#### Refinement

*R*[*F* ^2^ > 2σ(*F* ^2^)] = 0.043
*wR*(*F* ^2^) = 0.115
*S* = 1.05
2659 reflections
155 parameters
H-atom parameters constrained
Δρ_max_ = 0.18 e Å^−3^
Δρ_min_ = −0.20 e Å^−3^

### 

Data collection: *CrystalClear* (Rigaku, 2005 ▶); cell refinement: *CrystalClear*; data reduction: *CrystalClear*; program(s) used to solve structure: *SHELXS97* (Sheldrick, 2008 ▶); program(s) used to refine structure: *SHELXL97* (Sheldrick, 2008 ▶); molecular graphics: *SHELXTL* (Sheldrick, 2008 ▶); software used to prepare material for publication: *SHELXTL*.

## Supplementary Material

Crystal structure: contains datablocks I, global. DOI: 10.1107/S1600536808017613/rn2041sup1.cif

Structure factors: contains datablocks I. DOI: 10.1107/S1600536808017613/rn2041Isup2.hkl

Additional supplementary materials:  crystallographic information; 3D view; checkCIF report

## Figures and Tables

**Table 1:** Hydrogen-bond geometry (Å, °)

*D*—H⋯*A*	*D*—H	H⋯*A*	*D*⋯*A*	*D*—H⋯*A*
N3—H3*D*⋯N5^i^	0.86	2.11	2.934 (2)	162

Symmetry code: (i) 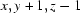.

**Fig. 1.**
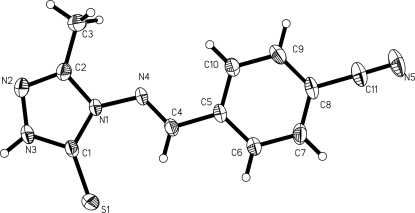
A view of the compound with the atomic numbering scheme. Displacement ellipsoids were drawn at the 30% probability level.

**Fig. 2.**
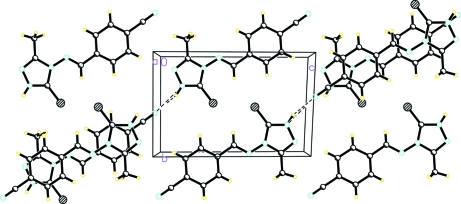
A packing diagram of the title compound, viewed down the a axis.

## supplementary crystallographic information

### Comment

It has been found that 1,2,4-thiadiazoles possess a broad spectrum of biological activities and can be widely used as fungicides (Sharma & Bahel, 1982) and insecticides (Grasso, 1988). In addition, amine- and thione-substituted triazoles have been studied as anti-inflammatory and antimicrobial agents (Eweiss *et al.*, 1986; Awad *et al.*, 1991). Benzotriazole and its derivatives comprise an important class of corrosion inhibitors, typically used as trace additives in industrial chemical mixtures, such as coolants, cutting fluids and hydraulic fluids (Pillard *et al.*, 2001). We present its crystal structure here. The molecule exists in the thione tautomeric form, with an S···C distance of 1.6752 (3) A °, which indicates substantial double-bond character for this bond [1.671 (24) A °, Allen *et al.*, 1987]. The dihedral angle between thione-substituted triazole ring and benzonitrile ring is 4.28 (3) °. N-H···N hydrogen bonds are observed in the crystal structure which link the molecules into dimers.

### Experimental

A mixture of 4-amino-3-methanyl-1*H*-1,2,4-triazole-5(4*H*)- thione (0.02 mol) and 4-formylbenzonitrile (0.02 mol) was refluxed at 391 K for 20 min in methanol. The mixture was then filtered and crystallized from ethanol to afford the target material (yield 89%). Single crystals suitable for X-ray measurements were obtained by recrystallization from ethanol at room temperature.

### Refinement

H atoms were calculated geometrically, with C—H distances in the range 0.93 to 0.97Å and an N—H distance of 0.86 Å, and refined using a riding model, with *U*_iso_(H) =1.5Ueq(C) for methyl H atoms and 1.2Ueq(C,*N*) for the other H atoms.

### Crystal data

**Table:**

C_11_H_9_N_5_S	*Z* = 2
*M**_r_* = 243.29	*F*_000_ = 252
Triclinic, *P*1	*D*_x_ = 1.371 Mg m^−^^3^
Hall symbol: -P 1	Mo *K*α radiation λ = 0.71073 Å
*a* = 6.975 (2) Å	Cell parameters from 1492 reflections
*b* = 7.682 (2) Å	θ = 3.0–27.4º
*c* = 11.412 (2) Å	µ = 0.26 mm^−^^1^
α = 90.262 (7)º	*T* = 293 (2) K
β = 94.328 (14)º	Block, colorless
γ = 104.713 (17)º	0.70 × 0.50 × 0.50 mm
*V* = 589.5 (3) Å^3^	

### Data collection

**Table:**

Rigaku Mercury2 diffractometer	2659 independent reflections
Radiation source: fine-focus sealed tube	2178 reflections with *I* > 2σ(*I*)
Monochromator: graphite	*R*_int_ = 0.022
Detector resolution: 13.6612 pixels mm^-1^	θ_max_ = 27.5º
*T* = 293(2) K	θ_min_ = 3.2º
CCD_Profile_fitting scans	*h* = −9→9
Absorption correction: Multi-scan(CrystalClear; Rigaku, 2005)	*k* = −9→9
*T*_min_ = 0.854, *T*_max_ = 0.901	*l* = −14→14
5954 measured reflections	

### Refinement

**Table:**

Refinement on *F*^2^	Secondary atom site location: difference Fourier map
Least-squares matrix: full	Hydrogen site location: inferred from neighbouring sites
*R*[*F*^2^ > 2σ(*F*^2^)] = 0.043	H-atom parameters constrained
*wR*(*F*^2^) = 0.115	*w* = 1/[σ^2^(*F*_o_^2^) + (0.0532*P*)^2^ + 0.1343*P*] where *P* = (*F*_o_^2^ + 2*F*_c_^2^)/3
*S* = 1.05	(Δ/σ)_max_ = 0.002
2659 reflections	Δρ_max_ = 0.18 e Å^−^^3^
155 parameters	Δρ_min_ = −0.20 e Å^−^^3^
Primary atom site location: structure-invariant direct methods	Extinction correction: none

### Special details

**Table:**

Geometry. All e.s.d.'s (except the e.s.d. in the dihedral angle between two l.s. planes) are estimated using the full covariance matrix. The cell e.s.d.'s are taken into account individually in the estimation of e.s.d.'s in distances, angles and torsion angles; correlations between e.s.d.'s in cell parameters are only used when they are defined by crystal symmetry. An approximate (isotropic) treatment of cell e.s.d.'s is used for estimating e.s.d.'s involving l.s. planes.
Refinement. Refinement of *F*^2^ against ALL reflections. The weighted *R*-factor *wR* and goodness of fit *S* are based on *F*^2^, conventional *R*-factors *R* are based on *F*, with *F* set to zero for negative *F*^2^. The threshold expression of *F*^2^ > σ(*F*^2^) is used only for calculating *R*-factors(gt) *etc*. and is not relevant to the choice of reflections for refinement. *R*-factors based on *F*^2^ are statistically about twice as large as those based on *F*, and *R*- factors based on ALL data will be even larger.

### Fractional atomic coordinates and isotropic or equivalent isotropic displacement parameters (Å^2^)

**Table:**

	*x*	*y*	*z*	*U*_iso_*/*U*_eq_	
C1	0.2635 (2)	1.3033 (2)	0.27180 (13)	0.0379 (3)	
C2	0.1665 (3)	1.0264 (2)	0.18636 (14)	0.0439 (4)	
C3	0.1029 (4)	0.8280 (3)	0.17603 (17)	0.0617 (5)	
H3A	0.0629	0.7922	0.0953	0.093*	
H3B	−0.0070	0.7840	0.2230	0.093*	
H3C	0.2115	0.7789	0.2030	0.093*	
C4	0.2745 (2)	1.1001 (2)	0.49268 (13)	0.0400 (4)	
H4	0.3196	1.2248	0.4992	0.048*	
C5	0.2738 (2)	0.9871 (2)	0.59672 (13)	0.0371 (3)	
C6	0.3299 (3)	1.0685 (2)	0.70682 (14)	0.0487 (4)	
H6	0.3678	1.1934	0.7142	0.058*	
C7	0.3300 (3)	0.9654 (3)	0.80588 (15)	0.0539 (5)	
H7	0.3652	1.0206	0.8798	0.065*	
C8	0.2773 (3)	0.7799 (2)	0.79417 (15)	0.0476 (4)	
C9	0.2227 (3)	0.6970 (2)	0.68453 (16)	0.0514 (4)	
H9	0.1879	0.5721	0.6772	0.062*	
C10	0.2200 (3)	0.8001 (2)	0.58632 (15)	0.0461 (4)	
H10	0.1821	0.7445	0.5127	0.055*	
C11	0.2800 (3)	0.6704 (3)	0.89609 (17)	0.0601 (5)	
N1	0.21505 (19)	1.11995 (17)	0.29285 (10)	0.0362 (3)	
N2	0.1847 (2)	1.1369 (2)	0.10000 (12)	0.0524 (4)	
N3	0.2429 (2)	1.3038 (2)	0.15394 (12)	0.0485 (4)	
H3D	0.2647	1.4017	0.1151	0.058*	
N4	0.2117 (2)	1.02188 (17)	0.39419 (11)	0.0385 (3)	
N5	0.2817 (3)	0.5822 (3)	0.97577 (16)	0.0811 (6)	
S1	0.32744 (8)	1.48037 (6)	0.36283 (4)	0.05691 (18)	

### Atomic displacement parameters (Å^2^)

**Table:**

	*U*^11^	*U*^22^	*U*^33^	*U*^12^	*U*^13^	*U*^23^
C1	0.0412 (8)	0.0381 (8)	0.0336 (8)	0.0090 (6)	0.0012 (6)	0.0115 (6)
C2	0.0541 (10)	0.0461 (9)	0.0328 (8)	0.0162 (7)	−0.0013 (7)	0.0027 (6)
C3	0.0905 (15)	0.0456 (10)	0.0494 (11)	0.0217 (10)	−0.0072 (10)	−0.0044 (8)
C4	0.0457 (9)	0.0390 (8)	0.0350 (8)	0.0100 (7)	0.0031 (6)	0.0111 (6)
C5	0.0366 (8)	0.0433 (8)	0.0325 (7)	0.0115 (6)	0.0048 (6)	0.0131 (6)
C6	0.0612 (11)	0.0433 (9)	0.0377 (8)	0.0070 (8)	0.0003 (7)	0.0109 (7)
C7	0.0647 (11)	0.0623 (11)	0.0325 (8)	0.0130 (9)	0.0007 (7)	0.0134 (8)
C8	0.0463 (9)	0.0592 (11)	0.0412 (9)	0.0186 (8)	0.0096 (7)	0.0259 (8)
C9	0.0628 (11)	0.0426 (9)	0.0525 (10)	0.0180 (8)	0.0112 (8)	0.0191 (8)
C10	0.0576 (10)	0.0443 (9)	0.0382 (9)	0.0160 (8)	0.0048 (7)	0.0102 (7)
C11	0.0646 (12)	0.0709 (13)	0.0517 (11)	0.0264 (10)	0.0144 (9)	0.0305 (10)
N1	0.0438 (7)	0.0370 (7)	0.0279 (6)	0.0105 (5)	0.0016 (5)	0.0089 (5)
N2	0.0719 (10)	0.0542 (9)	0.0308 (7)	0.0172 (8)	−0.0013 (6)	0.0065 (6)
N3	0.0652 (9)	0.0451 (8)	0.0328 (7)	0.0103 (7)	0.0016 (6)	0.0154 (6)
N4	0.0465 (7)	0.0389 (7)	0.0312 (6)	0.0125 (6)	0.0041 (5)	0.0136 (5)
N5	0.1056 (16)	0.0881 (14)	0.0603 (11)	0.0394 (12)	0.0213 (10)	0.0456 (10)
S1	0.0794 (4)	0.0363 (2)	0.0488 (3)	0.0070 (2)	−0.0080 (2)	0.00552 (19)

### Geometric parameters (Å, °)

**Table:**

C1—N3	1.3420 (19)	C6—C7	1.383 (2)
C1—N1	1.3887 (19)	C6—H6	0.9300
C1—S1	1.6546 (17)	C7—C8	1.382 (3)
C2—N2	1.296 (2)	C7—H7	0.9300
C2—N1	1.384 (2)	C8—C9	1.383 (3)
C2—C3	1.477 (2)	C8—C11	1.440 (2)
C3—H3A	0.9600	C9—C10	1.377 (2)
C3—H3B	0.9600	C9—H9	0.9300
C3—H3C	0.9600	C10—H10	0.9300
C4—N4	1.266 (2)	C11—N5	1.137 (2)
C4—C5	1.4735 (19)	N1—N4	1.3815 (16)
C4—H4	0.9300	N2—N3	1.371 (2)
C5—C6	1.384 (2)	N3—H3D	0.8600
C5—C10	1.391 (2)		
			
N3—C1—N1	101.52 (13)	C8—C7—C6	119.51 (17)
N3—C1—S1	127.16 (12)	C8—C7—H7	120.2
N1—C1—S1	131.32 (11)	C6—C7—H7	120.2
N2—C2—N1	110.55 (15)	C7—C8—C9	120.50 (15)
N2—C2—C3	126.08 (16)	C7—C8—C11	120.31 (18)
N1—C2—C3	123.37 (15)	C9—C8—C11	119.20 (18)
C2—C3—H3A	109.5	C10—C9—C8	119.79 (17)
C2—C3—H3B	109.5	C10—C9—H9	120.1
H3A—C3—H3B	109.5	C8—C9—H9	120.1
C2—C3—H3C	109.5	C9—C10—C5	120.33 (16)
H3A—C3—H3C	109.5	C9—C10—H10	119.8
H3B—C3—H3C	109.5	C5—C10—H10	119.8
N4—C4—C5	117.83 (15)	N5—C11—C8	179.2 (2)
N4—C4—H4	121.1	N4—N1—C2	118.04 (13)
C5—C4—H4	121.1	N4—N1—C1	133.14 (13)
C6—C5—C10	119.33 (14)	C2—N1—C1	108.80 (13)
C6—C5—C4	119.41 (15)	C2—N2—N3	104.07 (13)
C10—C5—C4	121.26 (14)	C1—N3—N2	115.04 (13)
C7—C6—C5	120.52 (17)	C1—N3—H3D	122.5
C7—C6—H6	119.7	N2—N3—H3D	122.5
C5—C6—H6	119.7	C4—N4—N1	120.55 (13)
			
N4—C4—C5—C6	−176.05 (15)	C3—C2—N1—N4	2.8 (2)
N4—C4—C5—C10	4.7 (2)	N2—C2—N1—C1	1.31 (19)
C10—C5—C6—C7	−0.9 (3)	C3—C2—N1—C1	−178.55 (17)
C4—C5—C6—C7	179.80 (16)	N3—C1—N1—N4	177.34 (15)
C5—C6—C7—C8	1.3 (3)	S1—C1—N1—N4	−3.4 (3)
C6—C7—C8—C9	−0.7 (3)	N3—C1—N1—C2	−0.99 (17)
C6—C7—C8—C11	178.86 (17)	S1—C1—N1—C2	178.25 (13)
C7—C8—C9—C10	−0.3 (3)	N1—C2—N2—N3	−0.99 (19)
C11—C8—C9—C10	−179.81 (17)	C3—C2—N2—N3	178.87 (18)
C8—C9—C10—C5	0.6 (3)	N1—C1—N3—N2	0.42 (19)
C6—C5—C10—C9	−0.1 (3)	S1—C1—N3—N2	−178.87 (13)
C4—C5—C10—C9	179.22 (16)	C2—N2—N3—C1	0.3 (2)
C7—C8—C11—N5	−176 (100)	C5—C4—N4—N1	−177.73 (12)
C9—C8—C11—N5	4(17)	C2—N1—N4—C4	172.61 (15)
N2—C2—N1—N4	−177.30 (14)	C1—N1—N4—C4	−5.6 (2)

### Hydrogen-bond geometry (Å, °)

**Table:**

*D*—H···*A*	*D*—H	H···*A*	*D*···*A*	*D*—H···*A*
N3—H3D···N5^i^	0.86	2.11	2.934 (2)	162

Symmetry codes: (i) *x*, *y*+1, *z*−1.
